# Understanding the impact of digital contact tracing during the COVID-19 pandemic

**DOI:** 10.1371/journal.pdig.0000149

**Published:** 2022-12-06

**Authors:** Angelique Burdinski, Dirk Brockmann, Benjamin Frank Maier

**Affiliations:** Institute for Theoretical Biology and Integrated Research Institute for the Life-Sciences, Humboldt University of Berlin, Germany; ISI Foundation: Fondazione ISI - Istituto per l’lnterscambio Scientifico, ITALY

## Abstract

Digital contact tracing (DCT) applications have been introduced in many countries to aid the containment of COVID-19 outbreaks. Initially, enthusiasm was high regarding their implementation as a non-pharmaceutical intervention (NPI). However, no country was able to prevent larger outbreaks without falling back to harsher NPIs. Here, we discuss results of a stochastic infectious-disease model that provide insights in how the progression of an outbreak and key parameters such as detection probability, app participation and its distribution, as well as engagement of users impact DCT efficacy informed by results of empirical studies. We further show how contact heterogeneity and local contact clustering impact the intervention’s efficacy. We conclude that DCT apps might have prevented cases on the order of single-digit percentages during single outbreaks for empirically plausible ranges of parameters, ignoring that a substantial part of these contacts would have been identified by manual contact tracing. This result is generally robust against changes in network topology with exceptions for homogeneous-degree, locally-clustered contact networks, on which the intervention prevents more infections. An improvement of efficacy is similarly observed when app participation is highly clustered. We find that DCT typically averts more cases during the super-critical phase of an epidemic when case counts are rising and the measured efficacy therefore depends on the time of evaluation.

## Author summary

Many countries relied on non-pharmaceutical interventions (NPIs) to mitigate or contain outbreaks during the COVID-19 pandemic. Because NPIs can have a dramatic socio-economic impact, policy makers were looking for less severe interventions to combat this global crisis. Since mid-2020, digital contact tracing (DCT) solutions have been implemented in many countries, raising the expectation that this intervention may effectively contain outbreaks without requiring more severe NPIs. Analyzing a stochastic infectious-disease network model that captures the essential elements of realistic contact structures, disease dynamics, NPIs, testing, and is based on empirical results regarding app adoption and usage behavior, we estimate that the intervention’s success with regard to the expected reduction of infections is on the order of single-digit percentages, mostly regardless of contact network structure. Only when contact networks either exhibit both high local clustering and a narrow degree distribution, or when app participation is highly clustered will DCT efficacy be increased.

## 1 Introduction

During the ongoing coronavirus disease 2019 (COVID-19) pandemic, the severe acute respiratory syndrome coronavirus type 2 (SARS-CoV-2) caused over 500 million confirmed infections and more than 6 million deaths worldwide up to June 23, 2022 [1]. Among other pivotal measures to mitigate or contain the disease’s spread, the most common one is testing and isolation of symptomatic individuals [2, 3]. While this intervention is usually effective, a proportion of transmissions in the COVID-19 pandemic occur from asymptomatic, paucisymptomatic, or presymptomatic infected individuals, which curbs its success [4–6].

A non-pharmaceutical intervention (NPI) that can help identifying non-symptomatic, yet infectious individuals is “contact tracing” (CT), where epidemiological relevant contacts of confirmed index cases are traced and isolated [2]. This procedure effectively shortens the infectious period of potentially infected secondary cases, reducing the number of tertiary infections. However, if the tracing mechanism takes too much time to identify and isolate contacts, only few transmissions are prevented [7], a problem that many countries face when their public health system is overburdened. With the intention to accelerate and supplement the manual tracing process, Bluetooth-based digital contact tracing (DCT) mobile phone applications (“apps”) were introduced in multiple countries over the course of 2020, for instance in the European Union [8]. These apps measure exposure to other individuals by using low-energy Bluetooth technology to identify other phones in close proximity that run the same or a compatible application. If tested positively, an index case can use the app to send notifications to potentially exposed individuals automatically who can then contact authorities, isolate themselves or get tested [9]. The major prospect of DCT as compared to manual CT was that infection chains might have been broken sufficiently fast to contain an outbreak [10]. In addition to the benefit of rapid notification upon case confirmation, and thus a reduced time until quarantine, DCT may also identify contacts that are unknown to the index case, an advantage compared to manual CT [11, 12]. The success of such large-scale digital mitigation strategies mostly depends, for a voluntary and decentralized approach, on acceptance in the general population, proper usage of the application, and technical properties [13].

By now, numerous studies have been conducted regarding benefits and limitations of DCT concerning its use during the COVID-19 pandemic [10, 14–24]. Early studies suggested that containment might be possible with high app participation [10, 14, 15] and assumed app participation rates of ≈ 50% as achievable or even as lower bounds [14, 16, 17]. Empirical results from Spain, Germany, and the UK suggest, however, that eventually participation rates of only ≈ 30% could be or were reached [11, 24, 25]. The fraction of sampled contacts scales quadratically with participation [10, 18, 22, 23]. So, for instance, if a randomly selected 50% of the population participates, approximately 25% of all contacts occur between pairs of individuals that are users of a DCT application, while only 9% of all contacts are traceable if 30% participate, therefore already greatly diminishing the potential effect of the intervention. Additionally, optimistic assumptions were made regarding technical properties of the apps and user behavior, e.g. zero or short delays between isolation of index case and tracing of secondary cases [14–18, 21], or high probability of tracing success per isolation event [14, 16, 18, 19, 22]. However, for instance in Germany, delays between isolation/testing of index cases and notification of a contact are expected to be larger (see Methods), while only 40% of users uploaded positive test results to the app [25]. Similarly, the success of the intervention relies on successful identification and isolation of index cases [10, 20, 22] and many studies assumed that around 50% or more of infected individuals would be identified/isolated and could potentially trigger digital contact tracing [14–16, 18, 19], yet ascertainment rates were suspected to be of lower value in many countries [26–28].

In general, modeling studies distributed app users randomly according to smartphone adoption rates in different age strata. It is, however, well known that behavior regarding compliance to mitigate public health crises is clustered, e.g. regarding intention to vaccinate [29, 30], which raises the question how DCT efficacy changes with high local clustering of app adoption.

A lot of the aforementioned parameter values and model design choices were based on assumptions, but by now, we can rely on empirical results that measured compliance and app usage, for instance in La Gomera [11], Switzerland [31], Norway [12], and Germany [25]. It was found that 64% (La Gomera, Spain), 60.3% (Zurich, Switzerland) or 72% (England and Wales, UK [24]) of all app users would or did upload a positive test result. This is in agreement with the findings in Norway where 50%–70% of app users were reported to be active daily and may therefore be categorized as active users. As mentioned above, in Germany, ≈ 40% of app users uploaded their positive test results up to June 23, 2022 [25]. Uploaded test results led to 6.3 (La Gomera), 4.3 (Zurich) or 4.2 (UK) notified individuals per index case. Of those notified contacts, 10% and 53% reached out to authorities for follow-ups in La Gomera and Zurich, respectively. Both studies concluded a population-wide app participation of around 30%. The proportion of notified contacts that are unknown to the index case ranged from 11% (Norway) to 20%–40% (La Gomera), thereby underlining the limited potential of DCT apps to identify unknown, i.e. random contacts.

The existence of these empirical results therefore warrants an analysis of the intervention’s efficacy in light of less optimistic assumptions.

Discussing the intervention’s potential for containment, many modeling studies concentrated on estimating epidemic growth rates or the effective reproduction number [10, 16, 17, 20, 21], yet containment was not achieved anywhere without falling back to other NPIs. In case of an uncontained outbreak, a way to measure an intervention’s mitigation potential is by counting the number of intervention-induced averted cases, or the relative reduction in outbreak size, also called overall attack rate. In that regard, given 30% app uptake, one study suggested that over a period of five months, relative reductions on the order of 20% can be reached in the state of Washington, US [15], stopping measurement before the end of the respective outbreaks. For the UK, a study suggested a relative reduction on the order of 15%, measured almost exclusively over a period of epidemic growth [24]. As contact-tracing may induce slow decay after reaching a peak [22], it is unclear how the time of evaluation influenced the measured mitigation potential in the studies cited above. Indeed, one study found that for France, a relative reduction in outbreak size of 7% can be expected for 30% app uptake when the respective outbreaks were measured until they ceased [19]. Measuring a reduction in outbreak size for an outbreak on two small, empirically recorded proximity contact networks resulted in values of ≲ 5% and ≈ 10%, respectively [18]. For Argentina, a study even suggested that DCT would have had virtually no effect [23].

We are therefore interested in finding out how the relative reduction of infections changes over time and how an efficacy measurement of only partial outbreaks influences the result.

While some studies assumed a well-mixed population [10, 24], or used empirically measured small-size contact networks [18, 20, 22], others explicitly modeled surrogate contact networks that have properties observed in real-world networks, i.e. high local clustering (for instance, households), small-worldness (low average shortest-path length between pairs of nodes), and broad contact number distributions (e.g. exponential) [14–16, 19]. However, no studies systematically investigated the influence of different contact structure properties on the success of DCT and hence, the influence of modeling choice regarding, e.g. homogeneous and random, or more realistic, locally clustered heterogeneous contact networks, remains elusive. Because drastic NPIs usually referred to as “lockdown” measures most likely changed both the population’s contact structure as well as its mixing properties during the pandemic, the question arises how these changes, contact network structure in general, and the distribution of app users might shape the success of DCT applications.

**Fig 1:**
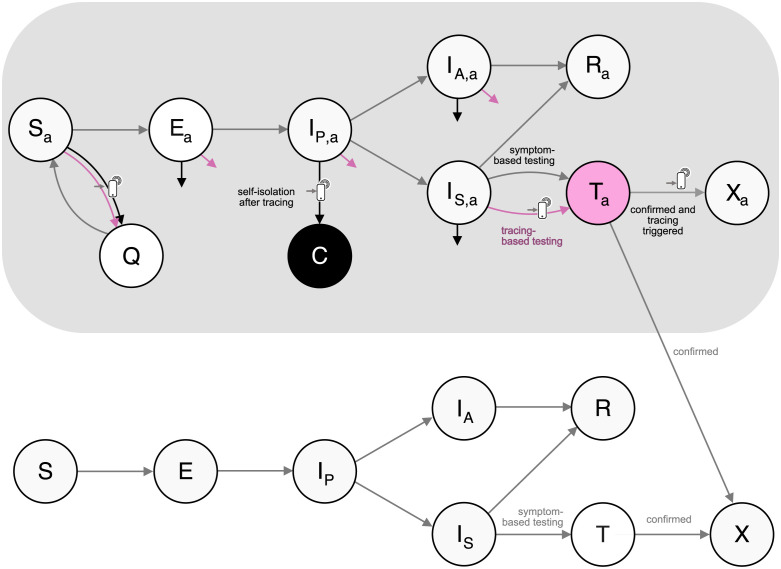
Schematic representation of the epidemic digital contact tracing model. App-participating individuals and groups are denoted by subscript *a*. Following an infection, susceptibles *S*_(*a*)_ become exposed *E*_(*a*)_ and enter a presymptomatic infectious state *I*_*P*(,*a*)_. Susceptible individuals can be infected by any neighbor in any infectious state (*I*_*A*(,*a*)_, *I*_*P*(,*a*)_, *I*_*S*(,*a*)_). After the presymptomatic phase, individuals become either asymptomatic *I*_*A*(,*a*)_ or symptomatic infectious *I*_*S*(,*a*)_ and can either recover *R*_(*a*)_ or, if symptomatic, they can be identified by symptom-based testing, i.e. enter *T*_(*a*)_, and are not infectious anymore. Tested non-app-participants (*T*) enter the final infected and quarantined state *X*. Tested app participants (*T*_*a*_) either upload their positive test result and enter *X*_*a*_, or not and enter *X*. The state transitions occur at rates listed in Table 1. When individuals upload the test result (*T*_*a*_ → *X*_*a*_) a series of conditional events is triggered. Infected app-participating neighbors (*E*_*a*_, *I*_*P*,*a*_, *I*_*A*,*a*_, *I*_*S*,*a*_) either self-quarantine *C* (black arrows) or get tested *T*_*a*_ (pink arrows), potentially inducing further tracing events. Susceptible neighbors always self-quarantine (*Q*) and return to the susceptible population afterwards.

**Fig 2:**
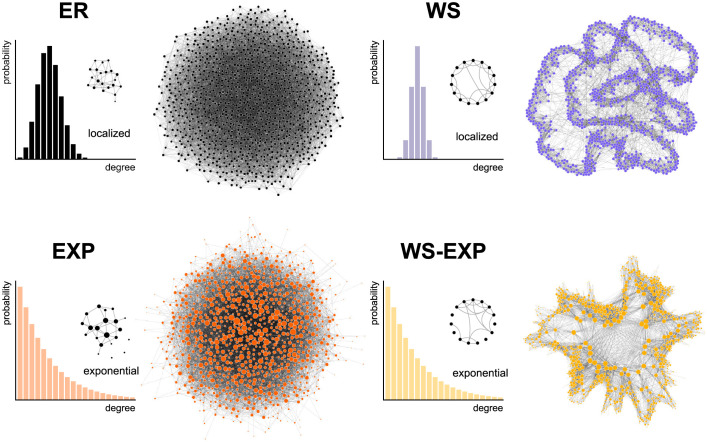
We analyzed the efficacy of DCT on four different network topologies: Erdős–Rényi networks (ER, low clustering and narrow (binomial) degree distribution), Watts–Strogatz–like small-world networks (WS, high clustering, narrow degree distribution), configurational model random networks with exponential degree distribution (EXP), and WS-like networks with high clustering and exponential degree distribution (WS-EXP).

To address the raised questions we devised a stochastic epidemic network model that combines the mechanistic aspects of DCT, e.g. app usage behavior of users that participate, app participation in general, etc. with the disease-dynamical components. The model permits a detailed analysis of the efficacy of DCT during the COVID-19 pandemic for empirically determined parameter regimes (see Fig 1 and Table 1) and a systematic investigation how DCT efficacy depends on parameters of the system. Because the impact of contact network structure, which cannot be directly assessed empirically on a large scale, remains elusive, we investigated DCT in four different network types, each alternating between exhibiting certain features that are generic for human contact networks, for instance a high degree of clustering and contact heterogeneity (see Fig 2), or the absence thereof, allowing a systematic comparison of network-structure influence.

**Table 1:** Events, parameters and description of the infectious disease model used in this study. Compartments of app participants are marked with subscript *a*. Events that are equal for app participants and non-users are marked with subscript (*a*), e.g. *A*_(*a*)_ → *B*_(*a*)_. Rates of events are displayed above the arrows.

Event	Parameter	Description
$S_{\left(a\right)}+I_{•}\overset{\phi_{•}}{\to }E_{\left(a\right)}+I_{•}$	*k*_0_ = 20, $R_{0}=2.5$, $\phi_{I_{A}}=\phi_{I_{S}}=\frac{\rho R_{0}}{2k_{0}}$, $\phi_{I_{P}}=\frac{\beta R_{0}}{2k_{0}}$	Susceptible individuals become exposed after infection by any of their infectious neighbors. To make simulations comparable across network models, we gauge the transmission rate per link using the well-mixed mass-action definition of the basic reproductive ratio $R_{0}$.
$E_{\left(a\right)}\overset{\alpha }{\to }I_{P(,a)}$	*τ*_*E*_ = *α*^−1^ = 3d [32, 34–38]	Exposed individuals become presymptomatic infectious at rate *α*, with an average latency period *τ*_*E*_.
$I_{P(,a)}\overset{x\beta }{\to }I_{A(,a)}$ $I_{P(,a)}\overset{(1-x)\beta }{\to }I_{S(,a)}$	*x* = 0.17 [39] *τ_P_* = *β*^−1^ = 2 d [32, 36]	After an average time *τ*_*P*_ a proportion *x* of presymptomatic infectious individuals become asymptomatic infectious and (1 − *x*) become symptomatic infectious at rate *β*
$I_{A(,a)}\overset{\rho }{\to }R_{\left(a\right)}$ $I_{S(,a)}\overset{\rho }{\to }R_{\left(a\right)}$	*τ_I_* = *ρ*^−1^ = 7 d [40, 41]	After an average infectious period of *τ*_*I*_, *I*_*A*_ and *I*_*S*_ individuals recover at rate *ρ* and are removed from the dynamics.
$I_{S(,a)}\overset{\kappa }{\to }T_{\left(a\right)}$	*q* = *κ*/(*κ*+ *ρ*) *q* ∈ {0.1, 0.3, 0.5}	As an alternative to recovery, symptomatic individuals can be detected and isolated at rate *κ*. *κ* is tuned via the detection probability *q*, i.e. the probability of detecting a symptomatic individual before the end of their infectious period and hence, quantifies the efficacy.
$T\overset{\chi }{\to }X$ $T_{a}\overset{z\chi }{\to }X_{a}$ $T_{a}\overset{(1-z)\chi }{\to }X$	*τ_T_* = *χ*^−1^ = 2.5 d *z* = 0.64 [11, 31]	Detected individuals enter the final infected and quarantined state at rate *χ*, after an average period *τ*_*T*_. Detected app users either upload a positive test result with probability *z* (entering *X*_*a*_) or not and behave as non-app-participants (entering *X*). Note that we also explore larger values of *χ* and *z*.
conditional stochastic events	*y* = 0.5 [31]	DCT occurs immediately with an individual triggering the event *T*_*a*_ → *X*_*a*_. We assume that all infected contacts of this individual are notified, a fraction *y* of which is tested and can trigger further tracing events (pink in Fig 1). The remaining fraction 1 − *y* self-isolate in group *C* (black in Fig 1). Susceptible neighbors are quarantined (entering *Q*) analogously to infected neighbors. Note that we also explore larger values of *y* and the impact of not quarantining susceptible individuals.
$Q\overset{\omega }{\to }S_{a}$	*τ*_*Q*_ = *ω*^−1^ = 10d [42]	Quarantined susceptible neighbors return to their normal behavior at rate *ω* after an average duration of *τ*_*Q*_.

Despite being less optimistic about usage behavior, our analysis suggests that in the absence of other NPIs, DCT might lead to a decrease in outbreak size on the order of single-digit percentages for realistic participation levels, which is in line with some of the more optimistic studies cited above [18, 19], but much smaller than other estimations [14, 15, 24]. We show that DCT has a high efficacy for large-scale outbreaks in networks that exhibit a high degree of clustering and a narrow degree distribution, suggesting enhanced efficacy when DCT is combined with NPIs that reduce contact heterogeneity, e.g. limiting the number of participants in larger-scale gatherings. However, if these contact reductions and reductions in contact heterogeneity lead to sub-criticality and containment, the relative efficacy of DCT decreases during those times. The increased performance of DCT in locally clustered networks also vanishes if the network’s degree distribution is moderately broad (here: exponential). We further find that the efficacy of DCT increases with increasing symptom-based testing or heterogeneous distribution of app participation. Moreover, super-criticality is crucial for DCT to work while introducing periodic NPIs can either increase or decrease its efficacy, depending on the time course of the pandemic. Optimizing key parameters enhances DCTs efficacy, the magnitude of which depends on app participation and the probability of identifying index cases before the end of their respective infectious period. Noticeably, we have to underline that our results disregard the high number of secondary cases that will have been known to index cases in anyway, which means our results present upper bounds in that regard. Consequently, we expect that DCT might help complementing manual CT processes and other interventions in times of rising case numbers, but should not be expected to suppress outbreaks substantially.

## 2 Methods

### Infectious disease model

To address the central questions around the efficacy of DCT, we designed a stochastic dynamic infectious-disease model that simulates spread on networks and is based on the generic susceptible-exposed-infectious-recovered/removed (*SEIR*) compartmental model [2], capturing the central mechanisms contributing to the outcome of DCT applications. The infectious state is split into subclasses to account for presymptomatic (*I*_*P*_), asymptomatic (*I*_*A*_) and symptomatic (*I*_*S*_) infectious individuals. We assume that 50% of transmissions are caused by presymptomatic individuals [4, 5, 32]. We also assume that asymptomatic and symptomatic individuals are equally infectious (while asymptomatic may be associated with lower viral shedding [33] they are less likely to change their behavior e.g. to reduce contacts compared to symptomatic individuals.

We introduce four additional compartments to capture symptom-based testing and DCT. As an alternative to recovery, symptom-based or DCT-induced testing detects symptomatic individuals or all infected app-using contacts, respectively. Detected individuals are isolated, hence not infectious anymore, and labeled *T*. Not every symptomatic individual is identified by symptom-based testing. Cases with mild symptoms may be less likely to seek medical help or tests may be limited when maximum testing capacity is reached. We denote the detection probability by *q* (see Table 1). Isolated individuals enter a final compartment *X* for documented, infected and isolated individuals. The tracing process is compiled in Table 1. Individuals remain in state *T* for *τ*_*T*_ = 2.5 days on average because (i) we assume the time between testing and receiving its result is between 1 to 2 days and (ii) uploading the test result and receiving a notification might also delay the process more than 24 hours. Uploading the test result probably occurs within ≤ 24 hours and DCT applications e.g. the “Corona Warn App” in Germany, are updating in 24 hour intervals, delaying the time at which another app user may receive the notification. Finally, individuals labeled *C* account for undocumented, infected and DCT-induced self-quarantined individuals, label *Q* represents susceptible, DCT-induced, self-quarantined individuals who protect themselves when receiving an exposure notification, see Fig 1.

The population is split in two classes, the app users (subscript *a*) and untraceable individuals. These groups interact via transmissions such that app users can infect non-users and vice versa. Only app users can trigger tracing-events on other app-using contacts. Events, parameters, and a detailed description of the model are compiled in Table 1 and in Fig 1.

### Network structure of contacts

Because the typical infectious period is on the order of a few days contact networks can be modeled as an effective, averaged medium [43]. We therefore implement static contact networks in the model (see Fig 2). Because of privacy policies, it is generically impossible to measure the complete contact networks and history using DCT applications. Yet, it has been shown that topological features of contact networks, especially broader degree distributions, clustering, and community structure can have an impact on contagion processes that unfold on these networks [44–46] and both features are generically observed in human contact networks [47]. In order to investigate the respective impact of broader degree distributions and clustering and to compare and quantify their effects to a generic, well-mixed system, we consider networks from four parsimonious models.

As a reference model we chose Erdős–Rényi (ER) random networks [48–50] with mean degree *k*_0_ and a narrow, binomial degree distributions. ER networks lack both, high degree variance and clustering. To capture the effects of clustering, we use Watts–Strogatz-like networks (WS) that retain the small-world property of the ER networks but exhibit a high clustering coefficient. The WS networks are constructed from a linear ring of connections with randomly rearranged long-range connections. Details of the construction are discussed in Refs. [51, 52]. The ratio of short and long-range connections is quantified by the redistribution parameter *β* = 10^−6^ that is chosen such that the random walk mixing time of a WS network is comparable to a reference ER network with the same mean degree. The clustering coefficient of the resulting WS networks is C=0.7.

To investigate the effects of greater degree variance we construct a random network with exponential degree distribution (EXP). We use a configuration model in which the degree of each node is chosen from an exponential distribution, nodes are linked randomly, and self-loops and duplicate links are removed [53]. Note that in comparative studies, broad degree distributions are frequently modeled using scale-free networks that instead of an exponential degree distribution exhibit a broader, power-law tail. We chose an exponential distribution here for two reasons. First, the impact of scale-free topology on dynamical processes on networks may change qualitatively compared to broad degree distributions with finite moments and it becomes difficult to assess what the impact of “broader than normal” could be. For instance in scale-free networks the classical epidemic threshold can be absent [45]. Secondly, physical proximity and contact networks are better captured by exponential degree distributions [54].

Finally, we analyze DCT on a highly locally clustered, exponential degree distribution network (WS-EXP) that combines the features of WS and EXP networks. Detailed network construction rules can be found in S1 Text.

For simulations we chose network sizes of *N* = 200,000 nodes with a mean degree, i.e. average number of contacts of *k*_0_ = 20. This value is motivated by the observed average number of 6.3 contacts per index case and ≈ 33% app participation in the La Gomera experiment. Although our results are robust when *N* is much smaller, the chosen network size reduces statistical fluctuations. We investigated DCT efficacy on all four network types. In order to compare different systems, the topological, static features must be linked to the dynamics of the contagion process. We gauged the infection dynamics by demanding identical per-link transmission rates across networks. The transmission rate per link is given by the well-mixed basic reproduction number $R_{0}$ per contact (with *k*_0_ being the number of contacts, constant for all networks) and per infectious period (*τ*_*P*_ for presymptomatic infectious, *τ*_*I*_ for symptomatic or asymptomatic infectious). By $R_{0}$ we refer to the definition of the expected number of secondary infections caused by an infected individual in an otherwise susceptible, well-mixed homogeneous population. Note that this gauging procedure and the implicit reference systems used in this manuscript does not incorporate the topological impact of complex network structure. Especially in structures that exhibit local clustering, the high probability of two neighbors of an index node being connected, too, reduces the force of infection: if an individual infects two neighbors and both of these neighbors are connected, too, then the number of tertiary infections these nodes can cause is reduced. Therefore, only in well-mixed systems will the epidemic threshold coincide with $R_{0}=1$, when $R_{0}=\phi k_{0}/\rho$. Network topology and degree variability generically shift the epidemic threshold [55–57]. For most scenarios below we consider dynamics in the super-critical regime in all networks.

For the stochastic simulations we use an adapted version of Gillespie’s algorithm to simulate the link- and node-mediated processes [58]. The model and simulations were implemented using the infectious disease modeling framework “epipack” [59]. All simulations were initiated with *I*_*P*,0_ + *I*_*P*,*a*,0_ = *N*/100 presymptomatic infectious individuals and a fraction *a* of app participants. If not stated otherwise, initially infected and app participants are distributed randomly in the network. For each parameter set and network type, 100 independent simulations were performed until the total event rate reached zero at time *t*_f_. We define the final outbreak size Ω of a run as the sum of the number of documented, isolated infected individuals *X*_∞_ = *X*(*t*_f_) + *X*_*a*_(*t*_f_), undocumented, self-isolated infected individuals *C*_∞_ = *C*(*t*_f_), and undocumented, non-isolated recovered individuals *R*_∞_ = *R*(*t*_f_) + *R*_*a*_(*t*_f_).

The under-ascertainment factor is defined by *UA* = Ω/*X*_∞_, i.e. the ratio of actual and documented infections. For constant detection probability *q* and variable app participation *a*, we refer to *UA*_0_ as the baseline under-ascertainment factor, i.e. in the absence of DCT. Note that we assume a uniform removal rate of *κ* + *ρ* for individuals that are in a symptomatic infectious state, i.e. do not have an explicit parameter to control for the time until detection. We demonstrate in S4 Text that this simplification has little influence on our main results.

Apart from symptom-based testing and DCT, we do not consider the influence of other specific pharmaceutical or non-pharmaceutical interventions on the under-ascertainment factor.

## 3 Results

**Fig 3:**
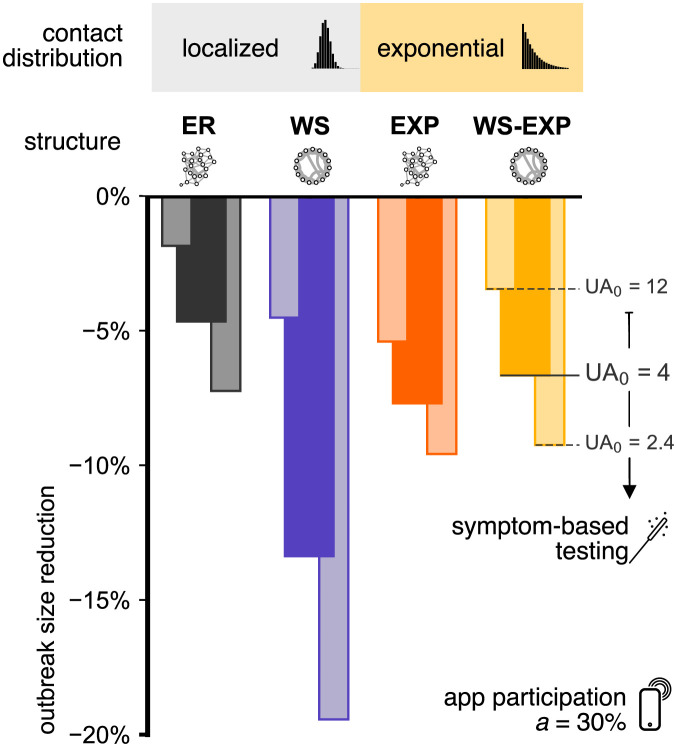
Relative mean outbreak size reduction 1 − 〈Ω(*a* = 30%)〉/〈Ω(*a* = 0)〉 caused by DCT in different network topologies. App participation was fixed at *a* = 30%, and symptom-based testing was assumed to lead to initial under-ascertainment factors of *UA*_0_ ∈ {12, 4, 2.4} (*q* = 0.1, 0.3 and 0.5, respectively). Note that 〈Ω(*a* = 0)〉 depends on the baseline under-ascertainment factor *UA*_0_ as well as the contact structure, see also Fig 7. DCT has a stronger effect in WS networks (purple) compared to the ER, EXP and WS-EXP networks. Increasing symptom-based testing (i.e. decreasing the under-ascertainment factor) enhances the efficacy of DCT.

Fig 3 depicts the reduction of outbreak size in all four representative networks with a selected app participation of *a* = 30% and for various selected values of the baseline under-ascertainment *UA*_0_. For an intermediate value *UA*_0_ = 4 (equivalent to a detection probability of *q* = 0.3 [26–28], see Fig A ii in S2 Text) we find that DCT alone leads to reductions in outbreak sizes on the order of ≈ 5 − 8%. The only network with larger effects (≈ 13%) is the WS network, indicating that high local clustering increases the effects of DCT. However, the positive impact of clustering disappears when highly clustered networks also exhibit an exponential degree distribution (WS-EXP). Since the positive effect of local clustering is already diminished by the introduction of an exponential degree distribution, we expect that for contact networks with an even broader degree distribution, DCT will not benefit from local clustering of contacts. Our model also permits capturing degree assortativity in the network which does not change the results (see Fig B in S1 Text). Therefore, we expect our results to hold for other networks that have the aforementioned properties.

Increasing the efficacy of symptom-based testing reduces the baseline under-ascertainment factor *UA*_0_ and enhances the relative success of DCT within the order of the single-digit percentage range. The under-ascertainment factor itself decreases with increasing app participation for a baseline under-ascertainment factor of *UA*_0_ = 12, otherwise it changes moderately (see Fig A ii in S2 Text), which suggests that DCT potentially contributes to a more precise assessment of the true extent of an outbreak when the detection probability is at the lower bound (*q* = 0.1) and the efficacy of the intervention will be less successful in any case. Furthermore, we find that the epidemic threshold is largely unaffected by DCT and results are unaffected by changing $R_{0}$ (see Fig A i in S2 Text).

It is natural to assume that app participation is not truly homogeneously distributed across different regions, age groups, or socioeconomic status and, consequently, the contact network of users. To assess the impact of heterogeneous app user distribution we analyzed non-random, clustered distribution of app participants and investigate the change in mitigation effects of DCT compared to randomly distributed app users. In general, when app participation is clustered, we expect that the fraction of links that connect two app participants and those that connect two non-participants have a higher proportion compared to a random distribution of app participants in the network, whereas links that connect one app participant with a non-participant are less frequent. Hence, we expect that the disease dynamics unfold differently in high-usage and low-usage regions of the network, bearing the question how this affects the system as a whole. If, for instance, we had two independent populations, one of size *aN* (app users) and of size (1 − *a*)*N* (not using the app), we would expect a linear relationship between app participation and outbreak size reduction: In the first group, DCT would mitigate the dynamics with maximized efficacy and in the second, an outbreak would reach its unmitigated size.

To distribute app participation in clusters we sample 1% seed nodes randomly and mark them as app participants. Next, we mark the seed nodes’ neighbors as app participants, their neighbors and so forth until an excess participation coverage of 10% is reached, i.e. *a* + 10%. The additional 10% of app participants is removed randomly to dilute the participant clusters. Note that this procedure is not meant to capture the clustered nature of app participation in real populations. Here we aim to compare the effects qualitatively and investigate the impact in different network topologies.

**Fig 4:**
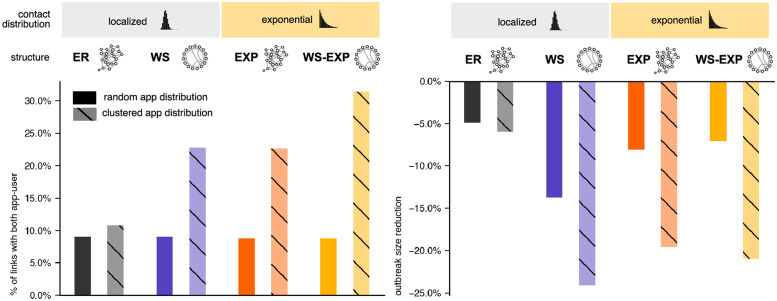
Percentage of links in the network with both nodes being app users (left) and relative outbreak size reduction caused by DCT with *UA*_0_ = 4 (*q* = 0.3) (right) in ER, WS, EXP, WS-EXP networks with an app participation of *a* = 30%.

Fig 4 depicts the impact of heterogeneous app participation on the change of DCT effectiveness in all four networks. As expected, inhomogeneous app participation changes the fraction of links that connect app users with other app users differently in all networks. For an app participation of 30% we expect ≈ 9% of links connecting users with other users in a well-mixed system. In the ER network, clustering does not change this fraction significantly, as expected. For WS (clustered network) and EXP (exponential degree distribution) we find a substantial increase to ≈ 22% and for the WS-EXP to ≈ 31%. Interestingly, this pure topological property, cannot be translated to the outbreak size directly, because of the combined effects of topology and disease dynamics. Heterogeneous app participation leads to a higher outbreak size reduction in all networks, except for the ER network. Note that, again, outbreak size reduction is largest in the WS network. As shown before, in a system with randomly distributed app participation, this advantage of local clustering was strongly diminished when, on top of local clustering, an exponential degree distribution was introduced (WS-EXP). In contrast, for a highly clustered app usage distribution, introducing higher degree variance on top of local clustering does not decrease DCT efficacy as strongly.

Many NPIs are aimed at reducing contacts and therefore possibilities for transmissions to occur. To shed light on the combined effects of DCT and contact-reduction-targeted NPIs (lockdown scenarios) we ran simulations in all four network topologies in which contacts and thus the corresponding well-mixed-system basic reproduction number were reduced by 40% (from $R_{0}=2.5$ to $R_{0}=1.5$). Topologically, this reduction is equivalent to changing the mean degree *k*_0_ from the original *k*_0_ = 20 to *k*_0_ = 12. Note that, a well-mixed basic reproduction value of 1.5 might correspond to a dynamic regime slightly below criticality in networks, see Fig A i in S2 Text, as explained in the Methods section. Nevertheless, in this regime outbreaks remain at a typical size for which the effects of DCT remain measurable. In fact, especially the dynamics in the vicinity of criticality may be of particular interest as systems of disease dynamics with inhibitory feedback of the host population naturally evolve into this critical regime [60, 61].

**Fig 5:**
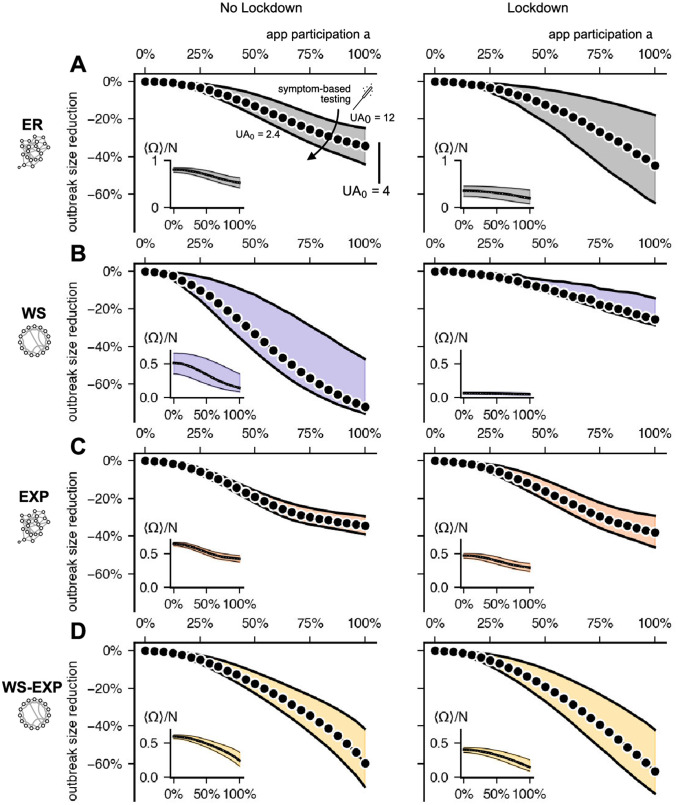
Capturing “lockdown” measures in our model, we compare the relative mean outbreak size reduction for (A) ER, (B) WS, (C) EXP and (D) WS-EXP networks in a situation where (left) the disease spreads freely to a situation where (right) the disease’s spread is mitigated by reducing the number of contacts by 40% while maintaining the transmission rate per link. Despite what our result of Fig 3 implies, the locally clustered structure does not enhance DCTs success when the disease is sub-critical. This suggests that super-criticality is necessary for DCT to work more efficiently.

Comparing no-lockdown and lockdown scenarios in ER, EXP, and WS-EXP networks (*k*_0_ = 20 and *k*_0_ = 12) we observe that reducing contacts while maintaining super-criticality can lead to a larger range of outbreak size reduction when increasing *a*, i.e. a smaller reduction for the lower bound of detection probability (*q* = 0.1, *UA*_0_ = 12) and larger reduction for the upper bound of detection probability (*q* = 0.5, *UA*_0_ = 2.4) (see Fig 5). Unmitigated dynamics lead to a stronger outbreak size reduction in WS networks (*k*_0_ = 20) compared to the other networks. The opposite is true during a simulated lockdown (*k*_0_ = 12): With the reduced number of contacts, the epidemic time course in the WS networks is sub-critical. Hence, a “lockdown” scenario in which contacts are reduced and clustering is more pronounced (a similar effect was observed in mobility networks [62]), implying sub-criticality, leads to smaller outbreak size reduction and suggests that super-criticality is required for effective DCT.

**Fig 6:**
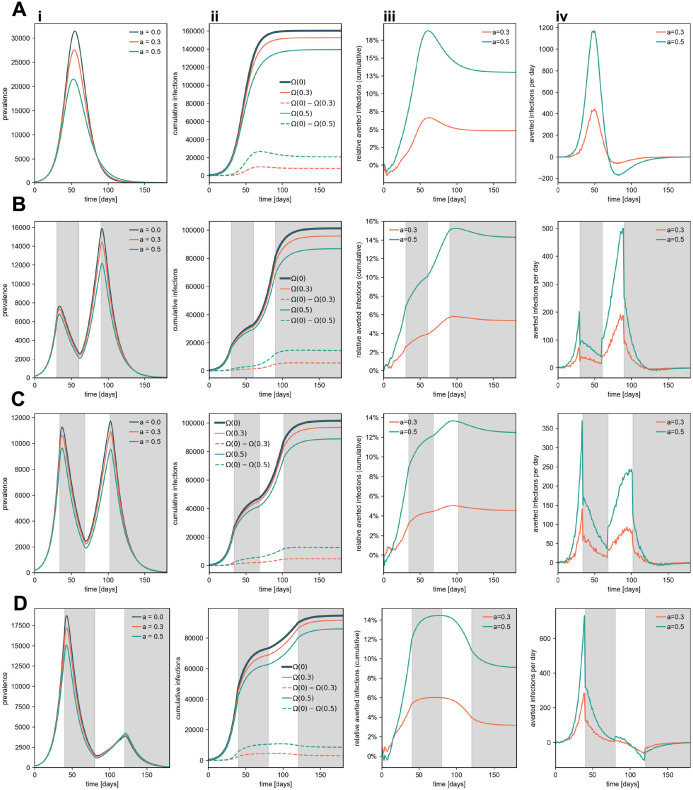
Comparison of (i) the prevalence, (ii) the cumulative infections Ω_*t*_(*a*) (and the difference between cumulative infections of “no DCT” and DCT-mitigated systems), (iii) the relative cumulative averted infections, and (iv) the prevented infections per day with *a* ∈ {0%, 30%, 50%} app participation and detection probability *q* = 0.3 (A) for spread without lockdowns and (B-D) for periodically introduced lockdowns where (B) *t*_1_ = 30d, (C) *t*_1_ = 34d, (D) *t*_1_ = 40d.

Many countries experienced complex patterns of epidemic time courses. Often, periods of partially mitigated spread were followed by suppressed growth, stifled by “lockdown” measures that temporarily reduced the effective reproduction rate. The interplay of seasonal effects, new variants and variability in behavioral responses lead to generic sequences of waves in incidence. To investigate how the time course of an epidemic influences the number of infections averted by DCT, we compared simulations in which (A) and epidemic unfolds for fixed external parameters with (B-D) a situation with alternating periods of lockdown and no-lockdown (see Fig 6). Specifically we investigated two consecutive lockdown scenarios. During lockdown periods, contacts were reduced by 60% and 50%, respectively. Between lockdowns, contacts were reset to the regular baseline level. At time *t*_1_ a lockdown began, at time *t*_2_ = 2*t*_1_, simulated restrictions were lifted, and at time *t*_3_ = 3*t*_1_, the second lockdown was initiated, this time without being lifted afterwards. We investigated three scenarios, *t*_1_ ∈ {30d, 34d, 40d}. For *t*_1_ = 30d, the second wave is larger than the first wave, for *t*_1_ = 34d, both waves reach similar magnitudes, and for *t*_1_ = 40d, the first wave is larger than the second, see Fig 6. For each scenario, we compared the relative outbreak size reduction if it was measured at time *t*, with respect to a scenario without DCT. This procedure mirrors the method that was used in the UK study to measure DCT efficacy during an outbreak [24]. We simulated 100 independent runs on ER networks (*N* = 200,000, *k*_0_ = 20, $R_{0}=2.5$, *I*_*P*,0_ = 0.001 × *N*). The results are depicted in Fig 6.

We found that the success of DCT depends sensitively on the phase of the pandemic time course and consequently on the point of time when efficacy would have been measured. Generically, we found that DCT prevents more cases in phases of epidemic growth. However, after an outbreak peaks naturally and incidences decline while no other NPIs mitigate the spread, prevalence may decay less quickly in DCT-mitigated systems. This may lead to a negative number of theoretically averted cases at this point of the outbreak, reducing the overall percentage of averted infections until the epidemic is over. This illustrates that using the method of [24] to measure the efficacy of DCT during rising incidences will naturally bias the result towards a higher relative number of prevented cases. This reduction in efficacy can be avoided when other NPIs suppress further growth of the pandemic, in which case the average number of averted cases can remain at higher values. This effect is more pronounced in systems where testing is not symptom-based (see Fig A in S3 Text).

Fig 6Ai depicts the time-resolved effects of DCT in a no-lockdown baseline simulation. With rising case numbers, the efficacy of DCT increases and more cases can be averted per day (compared to a system without DCT), both, relatively and absolutely. Because DCT has a higher efficacy in the super-critical regime the percentage of averted cases decreases with decreasing incidence, following the peak (c.f. Fig 6Aiii).

For *t*_1_ = 30d and 34d (see Fig 6B and 6C) DCT efficacy can be increased or maintained, for *t*_1_ = 40d, however, outbreak size reduction occurs in the epidemic’s sub-critical phase (see Fig 6D). This suggests that the influence of lockdowns on DCT efficacy is highly dependent on an epidemic’s time course and could explain differences of our results compared to other studies, the aforementioned UK study [24]. Note that in these simulations, efficacy only increases strongly when the prevalence attains high values.

**Fig 7:**
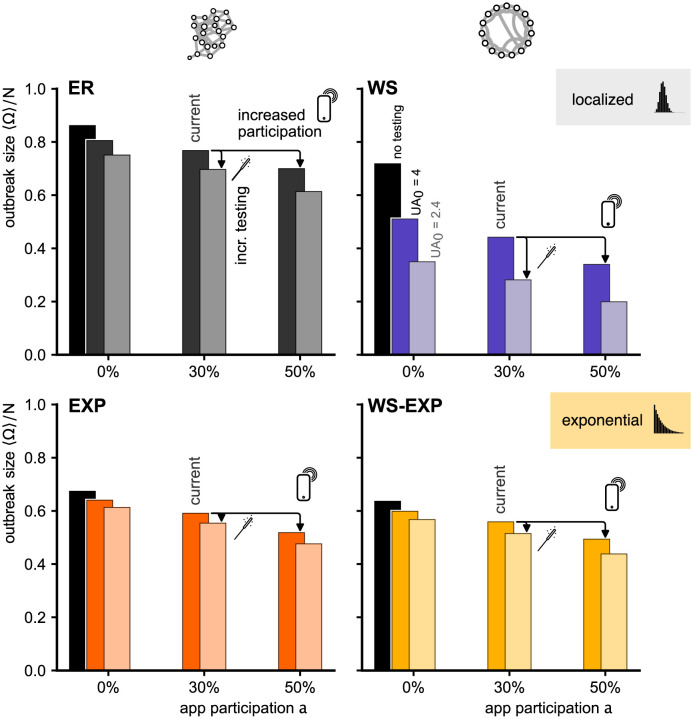
Outbreak size 〈Ω〉/*N* for different network models introduced in Fig 2, shown for app participation of *a* ∈ {0%, 30%, 50%}. We compared the absence of symptom-based testing, and testing that would lead to under-ascertainment factors of *UA*_0_ ∈ {4, 2.4} caused by *q* ∈ {0.3, 0.5}, respectively. Empirical observations suggest that several countries reached *a* ≈ 30% app participation and a under-ascertainment factor on the order of *UA*_0_ = 4 (marked as “current”) [25, 27, 28, 31]. We find no significant difference between increasing either symptom-based testing or app participation for three of the four network structures. For WS networks, an increase of symptom-based testing leads to a stronger reduction than an increase in app participation.

To analyze whether increasing app participation or increasing efficacy of symptom-based testing has a higher impact on mitigation we compared how much the outbreak size is reduced if either the under-ascertainment factor is reduced from *UA*_0_ = 4 to *UA*_0_ = 2.4 by detecting 20% more symptomatic individuals or the app participation is increased by 20% points from a baseline 30% to 50%. In ER, EXP, and EXP-WS networks neither increase has an advantage over the other (Fig 7). Only in the WS networks (locally clustered, narrow degree distribution), increasing symptom-based testing reduces the outbreak size more strongly.

**Fig 8:**
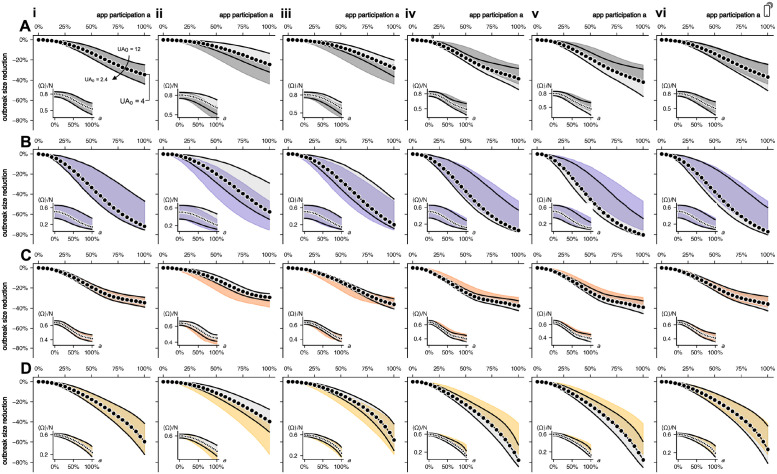
Outbreak size 〈Ω〉/*N* and relative outbreak size reduction caused by DCT with *UA*_0_ = 12 (upper boundary), *UA*_0_ = 4 (dotted) and *UA*_0_ = 2.4 (lower boundary) caused by *q* ∈ {0.1, 0.3, 0.5}, respectively, for increasing app participation *a*. Simulated on **(A)** ER, **(B)** WS, **(C)** EXP, and **(D)** WS-EXP networks with **(i)** the base parameter assumptions (in all subfigures highlighted in color for easier comparison), **(ii)** with only 50% of traced contacts reacting to a notification **(iii)** without isolation of susceptible contacts, **(iv)** where 100% (*y* = 1) of traced infected contacts can induce further tracing, **(v)** every app user uploads their result (*z* = 1) and, **(vi)** the delay of the event *T*_(*a*)_ → *X*_(*a*)_ is minimized (*χ* = 10).

Although we gauged model parameters based on a series of empirical studies to capture the situation of the COVID-19 pandemic, some parameters are difficult to quantify and subject to uncertainty and bias. Hence, we performed a systematic analysis of robustness and the model’s structural stability. We performed simulations and compared the results to the baseline scenario labeled (i) in Fig 8.

(ii) Only 50% of notified contacts react (instead of 100%). The baseline assumption that all notified contacts react, i.e. with either self-quarantine or with isolation and additional testing, reflects an optimal upper bound. We thus consider a 50% reduction such that 25% of notified individuals get tested and the other 25% choose to self-quarantine. Of all susceptible notified contacts 50% will self-quarantine (see Fig 8ii). As expected, this leads to a lower efficacy of DCT, i.e. lower outbreak size reduction.
(iii) Susceptible notified contacts will not self-isolate. In this case, we observe a smaller overall outbreak size reduction, especially for mid-range values of app participation (see Fig 8iii). However, non-trivial effects might arise in the regime of high participation, i.e. *a* → 1. Here we observe that, compared to the baseline, the reduction of outbreak size can be larger (WS-EXP). This can occur because if susceptibles do not self-isolate, case counts might be larger and the relative reduction of outbreaks increases, too, because DCT is more effective.
(iv) 100% of notified contacts get tested and hence induce further tracing (instead of 50%). This regime resembles a scenario equivalent to a situation of higher perceived risk and compliance to policies and sophisticated DCT technology and adaptation. For instance, in Vietnam, contact tracing was extended to 3rd-order contacts, all of which typically were tested [7], while values as low as 10% were reported as well (La Gomera [11]). We observe that the efficacy for ER and EXP networks is mainly increased for mid-range values of *a* while this is true for WS and WS-EXP networks at high values of *a* (see Fig 8iv).
(v) The delay *τ*_*T*_ = 2.5d between detection and notification of contacts is minimized to *τ*_*T*_ = (1/10)d. These simulations test the hypothesis of higher DCT efficacy by minimized time-delays. We increased the rate *χ* of the process *T*_(*a*)_ → *X*_(*a*)_ to *χ* = 10/d to represent an almost immediate upload of the test result (see Fig 8v). We observe for all networks an increased efficacy of DCT (see Fig 8v).
(vi) 100% of individuals upload their test result (instead of 64%). To increase the proportion of app users who upload their test result, we increased *z* from *z* = 0.64, which was found in empirical studies, to *z* = 1. We can see that the increase in efficacy is primarily visible at high values of app participation for the lower bound of the baseline under-ascertainment factor (*UA*_0_ = 2.4).

## 4 Discussion

Contrary to the positive expectations DCT has raised initially (see, e.g. refs. [10, 14, 15]), we conclude that our model, parameterized with less optimistic values, indicates that its impact on the reduction of COVID-19 outbreaks is rather supportive, which is in line with what was observed in the real world, where, for instance, no European country that introduced DCT was able to contain future outbreaks of COVID-19 without falling back to harsher NPIs. While our results suggest a relative reduction of case numbers in the single-digit percentages for otherwise unmitigated outbreaks, we ignored the fact that many of the cases that are found via DCT will be found via manual CT, as well (by authorities or by self-induced household isolation), as suggested by the empirical studies in Spain and Norway [11, 12].

On the other hand, we might have underestimated app usage clustering and overestimated under-ascertainment, which was recently observed to be lower in certain regions in Germany (but still on the order of a factor of four in many regions with larger outbreaks) [63]. Both effects increase DCT efficacy, as we have demonstrated. Furthermore, allowing positive antigene test results to trigger tracing both increases isolation probability and decreases delays until notification, which is nevertheless bounded by 24h due to the technical properties of the European app. Yet, we also demonstrated that short delays until notification led to minor changes in the results, and that for higher ascertainment of symptomatic individuals the outbreak-size reduction will still be within the single-digit regime. Additionally, a study in France that relied on higher ascertainment and shorter delays concluded a relative reduction on the order of 7% on top of the reduction induced by household isolation, which shows that the potential of the intervention is limited nonetheless [19].

In the UK, higher values of relative outbreak size reduction have been estimated [24], which might be attributed to a higher baseline user compliance than what we assumed based on other empirical studies, or the fact that the impact of DCT was measured exclusively over a period where cases mostly grew, which is when the relative effect of DCT will be stronger, as we have demonstrated. In particular, we showed that the timing of evaluating DCT efficacy matters, with the relative outbreak size reduction decreasing after a peak has been reached.

We showed that neither exponential degree distributions alone nor combined with high local clustering drastically affect our results. Curiously, only for contact networks that exhibit large local clustering in combination with narrow degree distributions do we see positive deviations from the baseline expected efficacy of DCT. This is interesting, because it has been shown that one of the most efficient NPIs during the pandemic was the reduction of group sizes in gatherings [64] which is equivalent to “narrowing the tail” of the degree distribution of contacts. One could argue that in combination with this particular NPI the efficacy of DCT might be larger than what would be expected. Yet, we have also shown that for epidemic outbreaks in which NPIs lead to containment and small outbreaks on these kind of contact structures, DCT contributes to a reduction of outbreak sizes in the single-digit percentages, which narrows the increased efficacy down to hypothetical scenarios in which NPIs limit large gatherings but do not lead to containment.

Our analysis demonstrates that DCT indeed prevents cases, even if this number is in the single-digit percentages. Since every prevented case is a life potentially spared, one may argue that its implementation is of use in any case. Additionally, DCT can have a benefit other than slowing down the spread of the disease, potentially reducing the harm of mortality due to earlier medical treatment of an infected person. Also, our analysis suggests that efficacy can be enhanced by increasing participation rates, clustering of app usage, randomized or symptom-based testing, the proportion of test results uploaded to the app, the proportion of contacted people that trigger next-generation tracing, as well as the introduction of strict NPIs that strongly suppress growth after an outbreak emerged. Although higher participation rates than those observed can be achieved, the number of people eligible for participation in DCT will be limited regardless [13].

We did not explicitly compare the impact of DCT in contrast to other NPIs (e.g. mask mandates) in our analysis which we leave for future research. Other NPIs and mask mandates in particular are expected to reduce average viral shedding and would therefore reduce $R_{0}$, which we found to not increase DCT’s impact on relative case number reduction (see Fig A in S2 Text), although it might increase for temporarily changing NPIs.

In summary, despite the promising outlook DCT applications triggered initially, our results indicate that at best they can support manual CT when outbreaks become large, but will not mitigate outbreaks substantially on their own. Policy makers should therefore keep in mind that if containment or large-scale mitigation of COVID-19 or similar diseases is a societal goal, a reliance on other NPIs will be necessary.

## Supporting information

S1 Text Construction of WS-EXP networks. (PDF)

S2 Text*UA* and varying $R_{0}$.(PDF)

S3 Text Influence of random testing during multiple waves. (PDF)

S4 Text Model with an explicit delay of detection. (PDF)
