# Supplementary material for: Understanding the impact of digital contact tracing during the COVID-19 pandemic
Source: PLOS Digit Health. 2022 Dec 6;1(12):e0000149. doi: 10.1371/journal.pdig.0000149 (PMC9931320; doi:10.1371/journal.pdig.0000149)
Supplement: S1 Text — (PDF) [file pdig.0000149.s001.pdf]

S1 Construction of WS-EXP networks

Angelique Burdinski[1*◕], Dirk Brockmann[1], Benjamin Frank Maier[1¶◕],

1 Institute for Theoretical Biology and Integrated Research Institute for the Life-Sciences, Humboldt University of Berlin, Germany

◕These authors contributed equally to this work. * burdinsa@hu-berlin.de
¶bfmaier@physik.hu-berlin.de

We developed a model that exhibits both the small-world / high clustering of WS-like networks with an exponential degree distribution. Networks of this type are constructed as follows. First N nodes are arranged on a ring and labeled n. Each node n is assigned a degree k_n chosen randomly from an exponential distribution. A number of k_n open link-stubs are "attached" to node n. Next, we iterate over all nodes in one of three orders: (i) by descending degree, (ii) by ascending degree, or (iii) randomly. For each node n we iterate over other nodes m on the ring, sorted by lattice distance to n, starting with the nearest node. If node m has open stubs available and n is not yet connected to m, a link between the nodes is established. This continues until all of n's stubs are connected. This is repeated for the next node in line.

If focal nodes n are iterated in descending order, hubs are connected first and will likely find locally available stubs that belong to nodes of small degree. Later, nodes with small degrees will have to connect to other low-degree nodes that are far away. Therefore, the structure is dominated by local connections and a small number of low-degree nodes will have non-local connections. The generated network will resemble a lattice, but nodes will have exponentially distributed degrees. Since high-degree nodes are connected first and to nodes with small degrees, degree assortativity will be negative. The probability that an edge connects two nodes at lattice distance d will be concentrated at low values of d. An empirical analysis reveals that a small amount of edges will connect to far-away regions, yet d will not reach values of maximum distance $N/2$ (see Fig A).

If focal nodes n are iterated in ascending order, low-degree nodes are connected first and "fill up" the local connections such that once high-degree nodes are connected, only long-range connections are possible. With high probability these will connect only to other high-degree nodes. Therefore, hubs will play a mixing role, connecting different regions of the network, while low-degree nodes will contribute a lattice-like, highly clustered structure. Degree assortativity will be positive. The probability that an edge connects two nodes at lattice distance $\geq d$ approximately follows d^{-1} (see Fig A).

If focal nodes n are iterated in random order, low-degree nodes are connected first with higher probability (because there are significantly more low-degree nodes). However, low-degree nodes will also connect last with higher probability. Hence, nodes of any degree will form a lattice-like structure, while nodes of any degree will play a mixing role. Degree assortativity will be close to zero. The probability that an edge connects two nodes at lattice distance $\geq d$ approximately follows d^{-1} (see Fig A).

Since social networks tend to have positive degree assortativity [1, 2], we choose to construct networks in the "ascending" order only. In Fig B, we compare a single result on these networks to networks that were created using the "random" order to find that degree assortativity makes no substantial difference.

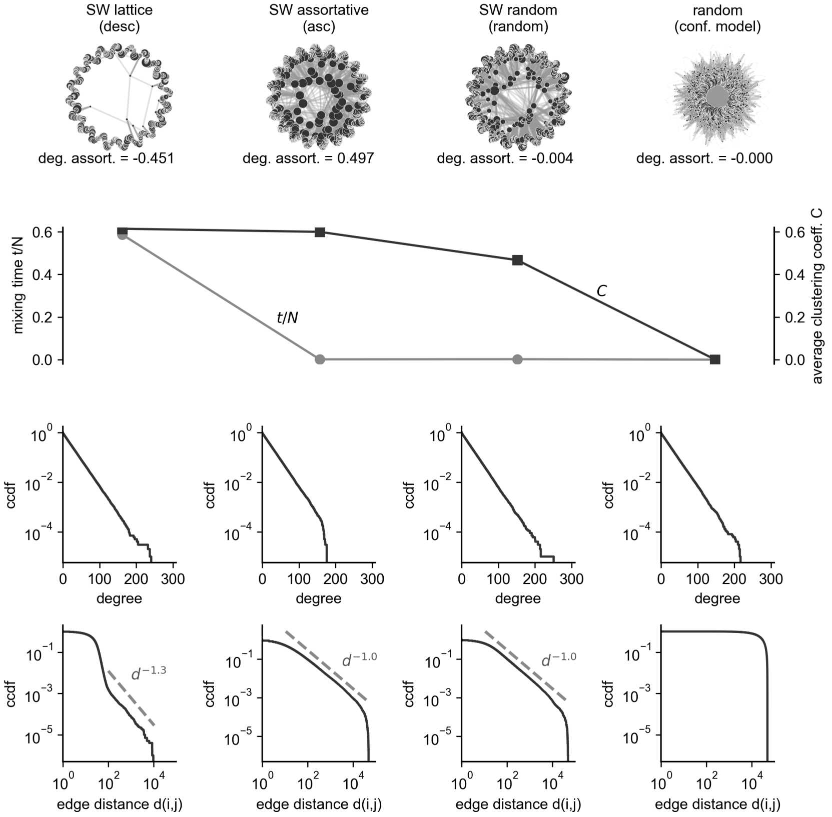

Fig A. Illustration of network properties of the WS-EXP model described above. Networks can be created by iterating over nodes in "descending", "ascending" or "random" order. We compare the structures to a well-mixed model with similar degree sequence (configuration model). The upper row shows illustrations of the networks that are constructed using the proposed algorithm. Node size represents degree and nodes are positioned closer to the center proportional to the maximum distance that any of its connecting links traverse. For these illustrations, networks with $N = 5000$ and $k_0 = 10$ were generated. For the rows below, a single network instance for each model was analyzed, with $N = 100{,}000$ and $k_0 = 10$. We use the per-node random walk mixing time of the network's largest connected component [3] and the average local clustering coefficient $\mathcal{C}$ [4] to illustrate the small-world effect. The value of $\mathcal{C}$ remains high for all models that are constructed this way. When iterating nodes in "descending" order, the mixing time is significantly larger than iterating in "ascending" or "random" order, both of which yield mixing times on the order of the random network (configuration model). For all network models, the degree distribution is approximately equal. For the "descending" model, links are mostly short distance, with a few links connecting regions that are further away. The remaining two models yield link distances d where the complementary cumulative distribution function (ccdf) follows a power-law d^{-1}. In contrast, the existence of an link does not depend on distance in the configuration model.

References

1. Newman MEJ. Assortative mixing in networks. Physical Review Letters. 2002;89(20):208701. doi:10.1103/PhysRevLett.89.208701.

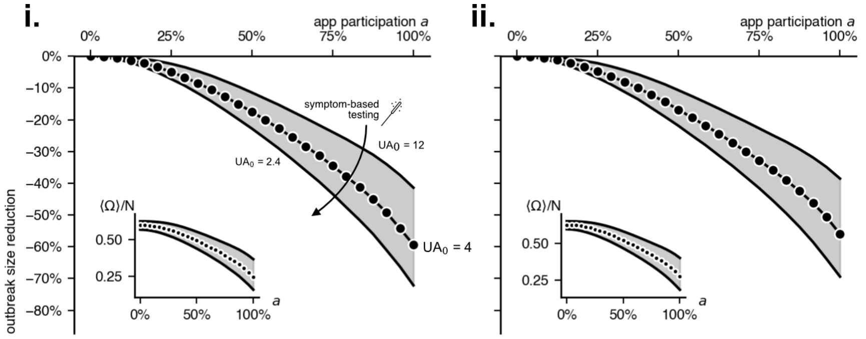

Fig B. Outbreak size $\langle\Omega\rangle/N$ and relative outbreak size reduction caused by DCT with $DF_0 \in \{12, 4, 2.4\}$ for increasing app participation a in **(i)** assortative small-world network (ordering: ascending) with exponential degree distribution and **(ii)** small-world network (ordering: random) with exponential degree distribution. Results between both models do not differ substantially.

2. Newman MEJ. Mixing patterns in networks. Physical Review E. 2003;67(2):026126. doi:10.1103/PhysRevE.67.026126.

3. Maier BF. Generalization of the small-world effect on a model approaching the Erdős–Rényi random graph. Scientific Reports. 2019;9(1):9268. doi:10.1038/s41598-019-45576-3.

4. Watts DJ, Strogatz SH. Collective dynamics of 'small-world' networks. Nature. 1998;393(6684):440–442. doi:10.1038/30918.
