# Supplementary material for: Understanding the impact of digital contact tracing during the COVID-19 pandemic
Source: PLOS Digit Health. 2022 Dec 6;1(12):e0000149. doi: 10.1371/journal.pdig.0000149 (PMC9931320; doi:10.1371/journal.pdig.0000149)
Supplement: S2 Text — (PDF) [file pdig.0000149.s002.pdf]

S2 *UA* and varying $\mathcal{R}_0$

Angelique Burdinski[1*◕], Dirk Brockmann[1], Benjamin Frank Maier[1¶◕],

1 Institute for Theoretical Biology and Integrated Research Institute for the Life-Sciences, Humboldt University of Berlin, Germany

◕These authors contributed equally to this work. * burdinsa@hu-berlin.de
¶bfmaier@physik.hu-berlin.de

To make sure that our analysis does not rely on the chosen basic reproduction number and to investigate whether DCT changes the epidemic threshold of the system we compared the mean and coefficient of variation (CV) of outbreak sizes for $\mathcal{R}_0 \in [0.1, 10]$. We compared $a = 0$ (no DCT) with $a = 30\%$ app participation (see Fig A i). For all (ER, WS, EXP, and WS-EXP) networks minor differences in mean outbreak size and no differences in outbreak size variation were observed for the entire range of $\mathcal{R}_0$. We conclude that the efficacy does not increase substantially for other $\mathcal{R}_0$ and that the epidemic threshold does not change with DCT.

Increasing the efficacy of symptom-based testing decreases the under-ascertainment factor but with increasing app participation the under-ascertainment factor shows only minor changes for both networks, except for high under-ascertainment factors like $DF_0 = 12$ (see Fig A ii). This suggests that increasing DCT does not contribute to obtaining a clearer picture of the outbreak. We attribute this finding to the fact that many contacts choose to self-quarantine instead of getting tested as described above.

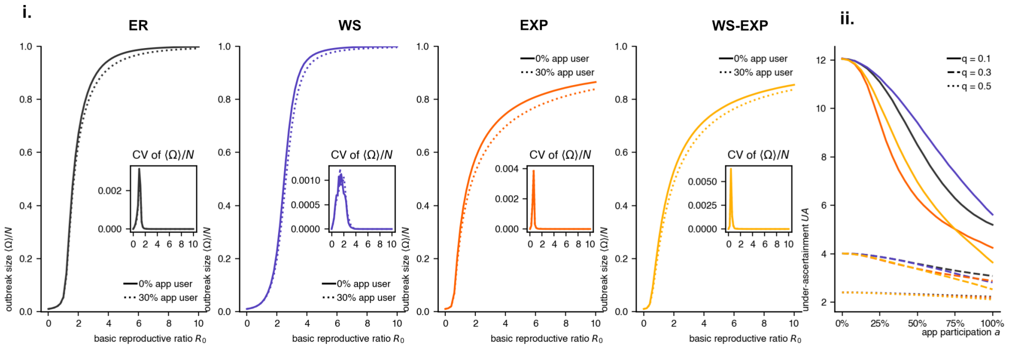

Fig A. Mean and coefficient of variation (CV) of outbreak sizes $\langle \Omega \rangle / N$ for increasing reproduction number $\mathcal{R}_0 \in [0.1, 10]$ with $DF_0 = 4$ and $a = 0$ (no DCT) or $a = 30\%$ app participation in the population on **(A)** ER, WS, EXP and WS-EXP networks **(B)** Under-ascertainment factors caused by increasing efficacies of symptom-based testing on (black) ER, (purple) WS, (orange) EXP and (yellow) WS-EXP networks.
