# Supplementary material for: Understanding the impact of digital contact tracing during the COVID-19 pandemic
Source: PLOS Digit Health. 2022 Dec 6;1(12):e0000149. doi: 10.1371/journal.pdig.0000149 (PMC9931320; doi:10.1371/journal.pdig.0000149)
Supplement: S3 Text — (PDF) [file pdig.0000149.s003.pdf]

S3 Influence of random testing during multiple waves

Angelique Burdinski[1*☯], Dirk Brockmann[1], Benjamin Frank Maier[1¶☯],

1 Institute for Theoretical Biology and Integrated Research Institute for the Life-Sciences, Humboldt University of Berlin, Germany

☯These authors contributed equally to this work. * burdinsa@hu-berlin.de
¶bfmaier@physik.hu-berlin.de

To analyze DCTs dependence on testing strategies, we additionally simulated a stripped-down version of our model where we do not differentiate between pre-, a-, and symptomatic infectious individuals (on Erdős-Rényi networks, see Fig A). This mimics "random" testing, where the probability to get tested does not depend on specific infection status. For simplicity, we also assume that (i) quarantining an individual leads to immediate notification of their contacts, (ii) every infected contact is immediately quarantined, (iii) no next-generation tracing is possible and (iv) susceptibles will not isolate themselves upon notification. We find a similar effect as described above for the epidemic that is only mitigated by testing: When case numbers rise, the relative number of averted cases increases. After reaching its peak, prevalence decreases slower in the DCT-controlled system than in the system without tracing, where herd immunity was reached. Hence, the total relative number of averted cases decreases again, reaching a value of 5% for $a = 0.3$ and $q = 0.3$ (c.f. Fig A 1). However, simulating an epidemic that has been suppressed by other NPIs twice, we find that high values of relative number of averted cases can be reached, that are then not reduced strongly when the outbreak is contained by a harsher reduction of the growth rate trough other interventions (see Fig A 2). From this, we conclude that the total efficacy of DCT can be kept higher when a larger outbreak is suppressed by the introduction of harsher lockdown measures (in combination with randomized testing).

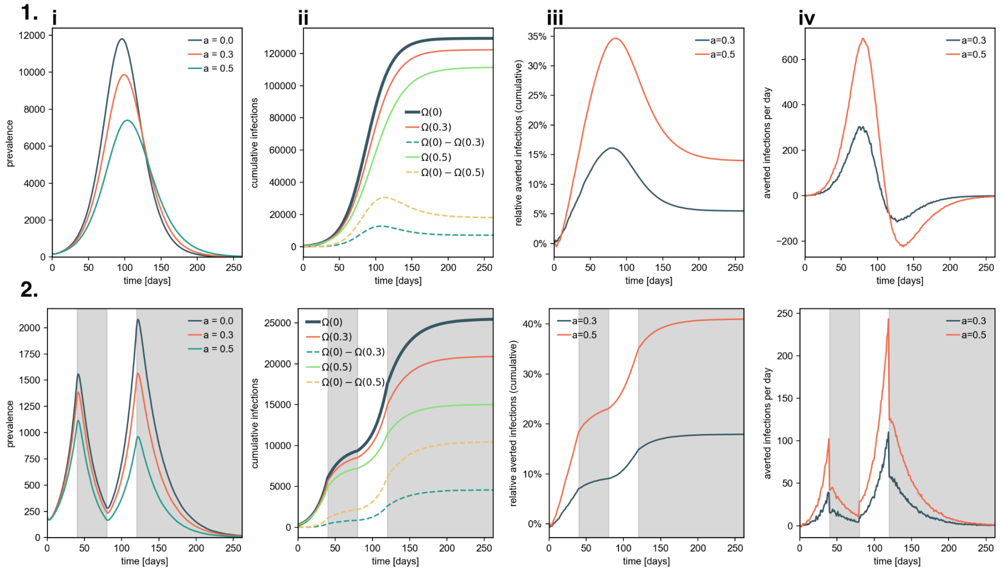

Fig A. A stripped-down version of our model where we do not differentiate between pre-, a-, and symptomatic infectious individuals. This mimics "random" testing, where the probability to get tested does not depend on specific infection status. We simulate epidemics that are **(1.)** only mitigated by quarantine and DCT as well as **(2.)** epidemics that are forced into two waves by other, abstract NPIs (lockdowns, for instance). We show **(i)** the prevalence, **(ii)** the cumulative infections $\Omega(t)$ (and the difference between cumulative infections of "no DCT" and DCT-mitigated systems), **(iii)** relative cumulative averted infections, and **(iv)** averted infections per day with $a \in \{0\%, 30\%, 50\%\}$ app participation. While for the (1.iii) otherwise unmitigated disease, DCT efficacy increases first and decreases afterwards, (2.iii) efficacy monotonically increases in the two-wave system. This illustrates both the influence of the specific trajectory of the epidemic as well as randomized testing. All curves are averages over 100 runs for each simulation. We exclusively simulated on Erdős–Rényi networks with $N = 200{,}000$ nodes and mean degree $k_0 = 20$.
