# Supplementary material for: Understanding the impact of digital contact tracing during the COVID-19 pandemic
Source: PLOS Digit Health. 2022 Dec 6;1(12):e0000149. doi: 10.1371/journal.pdig.0000149 (PMC9931320; doi:10.1371/journal.pdig.0000149)
Supplement: S4 Text — (PDF) [file pdig.0000149.s004.pdf]

S4 Model with an explicit delay of detection

Angelique Burdinski[1*◑], Dirk Brockmann[1], Benjamin Frank Maier[1¶◑],

1 Institute for Theoretical Biology and Integrated Research Institute for the Life-Sciences, Humboldt University of Berlin, Germany

◑These authors contributed equally to this work. * burdinsa@hu-berlin.de
¶bfmaier@physik.hu-berlin.de

In other models, the explicit *fast* identification and isolation of infectious individuals was shown to be a crucial factor in the success of digital contact tracing [1]. In order to minimize complexity in the original model, we did not include an explicit parameter to control for the time scale of identification, instead only relying on the isolation probability q from which the detection rate $\kappa = \rho q/(1-q)$ was fixed, which, in total, leads to an effective infectious period of $1/(\kappa+\rho)$ for either detection or undetected removal (recovery or self-isolation), see Fig A1.i. In the following, we investigate how our results change if an explicit short time passes until detection and isolation while isolation probability remains constant, see Fig A1.ii. To this end, we split the infectious symptomatic period into two infectious periods, one in which individuals reside until removal/recovery or detection, and one in which individuals that will not be detected reside until recovery while being infectious towards susceptible individuals. We fix the first time scale by introducing the detection time scale ζ and compute the detection rate $\kappa' = q\zeta$ as well as the first removal rate (non-detection) of $\rho_1 = (1-q)\zeta$. After non-detection, infectious individuals remain infectious for a time of $1/\rho_2$ and in order to obtain the same total mean effective infectious period as in the original model, we demand $1/\rho_2 = 1/\rho - 1/\rho_1$.

We compare the outbreak size reduction of both models for isolation probabilities $q = 0.3$ and $q = 0.5$, as well as varying app participation $a \in \{0.25, 0.50, 0.75, 1.00\}$, as well as a fast detection of $1/\zeta = 1\text{d}$. For each parameter combination, we ran 100 independent simulations on ER networks of $N = 10^4$ nodes with all remaining parameters being equal to the base case. The results are shown in Fig A2. We find that for realistic participation ratios ($a \leq 50\%$), the results only improve by a few percentage points, especially showing only marginal improvements for $a = 25\%$. The simulations suggest that faster removal seems to be of greater influence for high isolation probabilities and high app participation ratios, while not substantially changing the order of the outbreak size reduction.

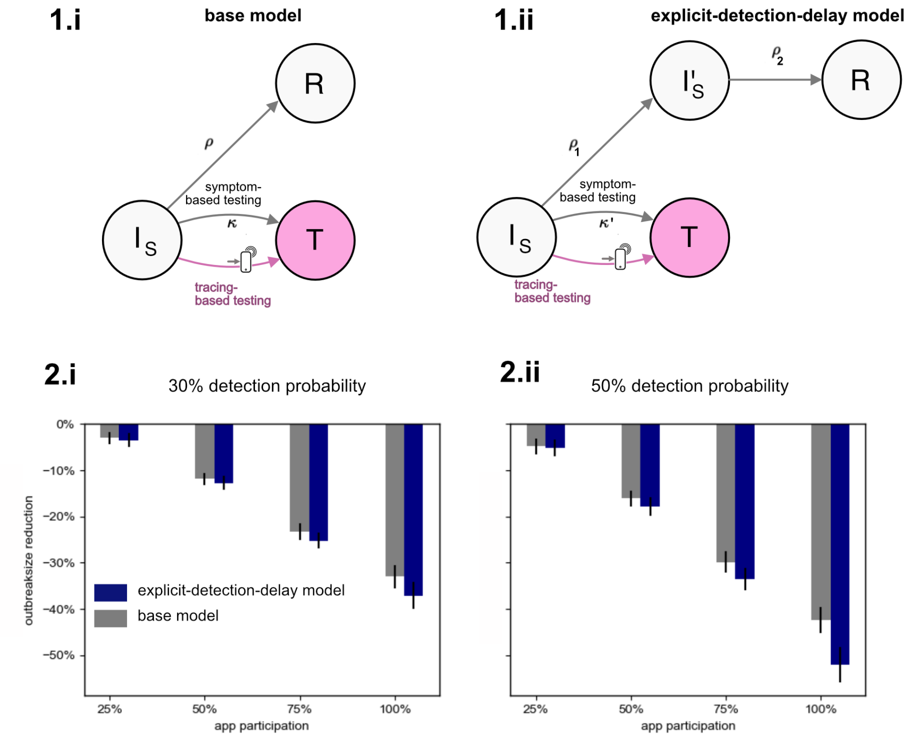

Fig A. The influence of faster isolation on the results. **(1.i)** Isolation dynamics that are being used in the original model. The assumption is that, on average, symptomatic infecteds may infect susceptible individuals on a time scale of $(\rho+\kappa)^{-1}$ until they either recover or are discovered and isolated. In total, a ratio of $q=\kappa/(\rho+\kappa)$ individuals are being detected, which fixes the isolation rate to $\kappa=\rho\frac{q}{1-q}$. **(1.ii)** In order to explicitly fix the time scale of isolation, we introduce the isolation rate ζ and demand that infected symptomatic individuals are removed/detected on a time scale of $1/\zeta$, with undetected individuals entering a second infectious period of duration $1/\rho_2$ during which they will not be detected anymore. A fraction $q=\zeta/(\zeta+\rho_1)$ will be identified and isolated, such that, in this variation of the model, the isolation rate becomes $\kappa'=q\zeta$ and the first removal rate is given as $\rho_1=(1-q)\zeta$. In order to have a constant total infectious period in both models, we fix $1/\rho_2=1/\rho-1/\rho_1$. **(2.)** Outbreak size reduction for both model variations. As in the main text we set $\rho=1/(7\text{d})$. Additionally, we demand a fast identification of $\zeta=1/\text{d}$ for the varied model. We set isolation probabilities of **(2.i)** $q=0.3$ and **(2.ii)** $q=0.5$ and show mean and standard deviation of 100 independent simulations on networks with $N=10,000$ nodes.

References

1. Ferretti L, Wymant C, Kendall M, Zhao L, Nurtay A, Abeler-Dörner L, et al. Quantifying SARS-CoV-2 transmission suggests epidemic control with digital contact tracing. Science. 2020;368(6491). doi:10.1126/science.abb6936.
